# The Impact of County Medical Community Reform on the Medical Service Efficiency of County-Level Public General Hospitals in China: A Case Study of Shanxi Province

**DOI:** 10.3390/ijerph192113827

**Published:** 2022-10-24

**Authors:** Yun Ye, Richard Evans, Li Jing, Muhammad Rizwan, Yan Xuan, Wei Lu

**Affiliations:** 1 School of Management, Hainan Medical University, 3 Xueyuan Road, Longhua District, Haikou 571199, China; 2 Faculty of Computer Science, Dalhousie University, Halifax, NS B3H 4R2, Canada; 3 Department of Health Management, School of Medicine and Health Management, Tongji Medical College, Huazhong University of Science and Technology, Wuhan 430030, China; 4 School of Economics and Management, Yangtze University, Jingzhou 434023, China; 5 Hainan Women and Children’s Medical Center, Haikou 570312, China

**Keywords:** county medical community reform, county-level public general hospitals, medical service efficiency

## Abstract

China introduced the county medical community (CMC) reform, aimed to provide high-quality medical resources to rural citizens, in 2017. This study examines the impact of the reform on the medical service efficiency of county-level public general hospitals in Shanxi Province, China. In total, 92 county-level public general hospitals from Shanxi Province were taken as the research objective, and the super-efficiency SBM-DEA model was applied to measure medical service efficiency. Further, a two-way fixed-effect model was used to evaluate the impact of CMC reform on the medical service efficiency of county-level public general hospitals by using health statistics data from 2014 to 2018. The study reveals that the CMC reform improved the medical service efficiency of county-level public general hospitals by 15.6%. Moreover, the CMC reform had regional heterogeneity in its impact on the medical service efficiency of county-level public general hospitals. The CMC reform improved the medical service efficiency of hospitals in the southern region more than in the northern region of the province. The medical service efficiency of hospitals in the central region was also improved by CMC reform, but the causal relationship was not found significant. Further, hospital-level factors (e.g., fixed assets, hospital stay, and regional health center) and environmental factors (e.g., GDP, population, urbanization rate, and government subsidies) affected the medical service efficiency of county-level public hospitals during the process of promoting the CMC reform.

## 1. Introduction

China introduced major healthcare reform in 2009 with the aim of providing citizens with equal access to basic healthcare across the country. Since it was introduced, the healthcare conditions of primary medical institutions have improved significantly [1]. Long-term weaknesses in primary medical services have gradually changed, while fairer distribution and accessibility to basic medical and healthcare services have significantly improved. However, problems still exist in China’s primary medical institutions with, for example, insufficiencies in quality medical resources, unreasonable structures, and uneven distribution of resources [2]. How to solve these problems remains a major concern.

Global experiences have proven the effectiveness of integrated healthcare delivery systems in solving the above problems [3]. The medical community is a form of such a system, being an effective exploration and practice mode to optimize the allocation of medical resources and improve the service level of primary medical institutions [4,5]. In 2017, the General Office of the State Council of China issued their guiding opinions on promoting the construction and development of medical communities [6], which proposed the construction of county medical communities in rural China. The aim behind the establishment of the CMC was to provide high-quality medical resources to rural citizens while exploring integrated county and township health management based on county-level hospitals, township hospitals, and village clinics. This involved the formation of a labor and cooperation mechanism between county, township, and village medical and health institutions to develop a coordinated, three-level county medical service system. Many provinces and cities in China have explored various CMC modes in rural areas [7,8].

County-level public general hospitals operate as the leader of primary medical institutions in China and provide medical services to rural residents. However, due to the unreasonable distribution and utilization of health resources between urban and rural areas, patients residing in rural areas prefer city-level hospitals rather than county-level public general hospitals [9,10]. For this reason, with many patients flowing out of the county, county-level public general hospitals often stand idle on a large scale, and medical resources are wasted [11]. One of the goals of the CMC reform is to improve the medical service efficiency of county-level public general hospitals and to keep patients in the county. Accordingly, it is crucial that we identify whether the efficiency scores of county-level public general hospitals improved by following the implementation of the CMC reform. Moreover, the heterogeneity of policy implementation effects should also be considered. Different regions have varying geographical, economic, and social development conditions and health resources. Therefore, it is also necessary to analyze whether the impact of the CMC reform on county-level public general hospitals in different regions is consistent to propose more precise policies and prevent “one size fits all” policies.

The aim of this study is to examine the impact of the CMC reform on the medical service efficiency of county-level public general hospitals in Shanxi Province, China. The main contributions of the study include that (1) we measure the efficiency of county-level public general hospitals in Shanxi Province and evaluate the changes in their medical service efficiency before and after the CMC reform; (2) we explore the impact of the reform on the medical service efficiency of county-level public general hospitals and analyze whether the relationship between the reform and efficiency significantly differs across regions; and (3) we provide pertinent policies and suggestions for the construction of the CMC in rural China.

Shanxi Province was selected as the research objective in this study, providing relevant information and policy implications for regional and national CMC reform. The reasons behind the selection of this area are: (1) The county population of Shanxi Province is 25.48 million, accounting for more than 73% of the total population [12], and the structural contradiction of county health resources is very prominent. How to improve the medical service efficiency of county-level public general hospitals and ensure that “serious diseases do not leave the county” is an urgent problem to be addressed in Shanxi Province. By taking Shanxi Province as the research object, it can provide suggestions for optimizing the reform of CMC and improving the medical service efficiency of Shanxi Province; (2) Shanxi Province is an economically underdeveloped area with a gross domestic product (GDP) per capita of CNY 50,528 in 2020 [12], ranking it 26 among 31 provinces and municipalities in mainland China. However, since the implementation of the CMC reform, it has made considerable improvements. For example, the province was selected as one of the ten new measures of “promoting medical reform and serving people’s health” in 2017, and the “Shanxi model” of comprehensive medical reform at county level was created, which was promoted to the whole country by the National Health Commission. Moreover, the province was selected as one of the two provincial-level pilot provinces to promote the construction of compact CMC in 2019 [13]. Therefore, taking this province as the research objective can provide experiences for other economically underdeveloped regions in the national context and globally to promote the CMC reform and optimize the allocation of medical resources in rural areas; and (3) Shanxi Province began to implement the CMC reform in 2016, which was far earlier than other provinces. This provides us with greater long-term samples than other provinces.

The remainder of this paper is organized as follows: Section 2 provides an overview of relevant research literature. Section 3 introduces the method and data in detail. Section 4 presents research results. Section 5 reports the discussion. Conclusions and limitations are presented in Section 6.

## 2. Literature Review

The county medical community is considered a form of integrated medical care with extant research on the topic focusing on two main aspects.

First, scholars have explored the performance evaluation of integrated medical care and county medical communities. For example, Guezennec [14] constructed an evaluation index system for the integrated healthcare service system that operates in Europe. The scheme aimed to be patient-centered, considering service accessibility, continuity, coordination, patient experience, and orientation in the community and its management. Mengxi et al. [15] took Deqing County in Zhejiang Province as a case and created a three-dimensional evaluation index system for system construction, institutional development, and social responsibility to evaluate the effects on county medical communities. He et al. [16] found that the implementation effects of county medical communities experienced in eastern, central, and southern China were found to be better. In contrast, the most unsatisfactory results were experienced in northeastern China. Sun et al. [17] selected 200 county-level hospitals as a sample and found that 51.5% of hospitals achieved unified procurement of medicines, 32% of hospitals achieved unified rules and regulations, and 72% of hospitals issued a medical community implementation plan. At the same time, 60.5% of hospitals introduced a medical community assessment mechanism. The overall promotional effect of the county medical community was considered better than previously experienced. Zhang et al. [18] found that the hospitals making the best progress in county medical community reform were those that were government-led; in such cases, the government empowered medical communities although less attention was paid to improving service capabilities and quality.

Second, studies have also focused on the impact of integrated medical care and county medical community reform on medical institutions and their patients. For instance, Greaves [19] found that the intervention effect of the Northwest London Integrated Care Pilot (NWLICP) reduces unnecessary health services and medical expenditures, improves clinical output and medical quality, and enhances the satisfaction of patients and medical staff. Similarly, Mastellos [20] evaluated the NWLICP through a questionnaire survey and found that integrated healthcare services can improve both service quality and patient experiences. Based on a study of seven county-level public hospitals, Yuan et al. [21] found that through participation in the county medical community reform, hospitals improved their service efficiency and capability. More patients were retained, and control of them was improved to a certain extent. Based on a study of thirteen district and township public health institutions, Liu et al. [22] found that, after participating in county medical community reform, the number of discharged patients from district-level medical institutions significantly reduced after the reform. Similarly, average outpatient expenses decreased after the reform, while the average hospital stay for patients admitted to township hospitals increased after the reform. Finally, per capita hospitalization expenses increased.

As may be seen from extant studies, empirical research into the impact of CMC reform in primary medical service institutions is relatively scarce [23]. Although some of the literature has studied the effects of CMC reform, it has mainly focused on the perspective of medical resource utilization—there are few studies on the medical service efficiency of county-level public general hospitals. This paper focuses on evaluating the efficiency of county-level public general hospitals in China before and after CMC reform. The empirical study objectively evaluated the effect of CMC reform and provided constructive references for policy makers and hospital managers.

## 3. Methods and Data

### 3.1. Method

#### 3.1.1. Efficiency Evaluation Method

We applied the super-efficiency slack-based measure–data envelopment analysis (SBM-DEA) model to measure the medical service efficiency of county-level public general hospitals. Compared with the traditional radial data envelopment analysis (DEA) models, such as CCR and BCC, the SBM-DEA model considers the influence of relaxation variables, which can reduce the deviation of efficiency evaluation. Tone [24] proposed the slack-based measure (SBM) model (the farthest distance function to the frontier), which solves the problem of the radial model not including relaxation variables in the measurement of inefficiency. The non-guided SBM model is detailed as follows:

(1) $$\mathrm{minimize}\;\rho =\frac{1-\frac{1}{m}\sum_{i=1}^{m}\frac{s_{i}^{-}}{x_{ik}}}{1+\frac{1}{q}\sum_{r=1}^{q}\frac{s_{r}^{+}}{y_{rk}}}\;\mathrm{subject}\;\mathrm{to}\;x_{k}=x\lambda +s^{-},y_{k}=y\lambda -s^{+},\lambda ,s^{-},s^{+}\geq 0$$

where *m* and *q* represent inputs and outputs, respectively, of each county-level public general hospital; $x_{ik}\;\mathrm{and}\;y_{rk},$ respectively, represent the ith input and the *r*th output of the *k*th county-level public general hospital. Furthermore, $s^{-}$ and $s^{+},$ respectively, represent the slack variable of the *i*th input and the slack variable of the *r*th output, whereas $\frac{s_{i}^{-}}{x_{ik}}\;\mathrm{and}\;\frac{s_{r}^{+}}{y_{rk}}$ represent the inefficiencies of the *i*th input and *r*th output, respectively; $x_{k}$ and $y_{k},$ respectively, represent the input and output of the *k*th county-level public general hospital. λ represents the adjustment matrix; xλ and yλ, respectively, represent the input and output of the frontier production line. The value of ρ is between 0 and 1. When ρ = 1, it indicates that the county-level public general hospital is highly effective and located at the efficiency frontier, and each slack is 0. When ρ is close to 0, it indicates that the county-level public general hospital is inefficient.

The super-efficiency SBM model is generated based on the SBM model. Compared with the SBM model, the super-efficiency SBM model can further compare and distinguish the efficient decision-making units (DMUs) at the frontier [25]; that is, if DMUs is evaluated as SBM strongly effective in Equation (1), i.e., ρ = 1, then its super efficiency is defined as:

(2) $$\mathrm{minimize}\;\rho =\frac{\frac{1}{m}\sum_{i=1}^{m}\frac{{\bar{x}}_{ik}}{x_{ik}}}{\frac{1}{q}\sum_{r=1}^{q}\frac{{\bar{y}}_{rk}}{y_{rk}}}\;\mathrm{subject}\;\mathrm{to}\;\sum_{j=1}^{n}x_{ij}\lambda_{j}\leq {\bar{x}}_{ik},\sum_{j=1}^{n}y_{rj}\lambda_{j}\geq {\bar{y}}_{rk},x_{ik}\leq {\bar{x}}_{ik},y_{ik}\geq {\bar{y}}_{ik},\lambda_{j},{\bar{y}}_{rk}\geq 0$$

Based on the input-output index of the evaluation of medical service efficiency in existing medical institutions [26,27,28,29], the number of licensed physicians, the number of registered nurses, the number of beds, and the number of equipment above ten thousand RMB were selected as input variables. At the same time, the number of outpatient visits, the number of emergency visits, total number of discharged patients, and the number of surgical patients were selected as output variables (see Table 1).

#### 3.1.2. Policy Effect Evaluation Method

In this paper, a two-way fixed-effect model is used to estimate the impact of the CMC reform on the efficiency of the medical services delivered by county-level public general hospitals while adding both individual fixed effects and time fixed effects to the model. Compared with the traditional panel model, the two-way fixed-effect model can eliminate the endogenous problems caused by missing variables. Variables that may be missed include unobservable factors at the individual hospital level that do not change over time but are different for each individual hospital (e.g., the internal operating conditions of the hospital, etc.) and unobservable factors at the time level that change over time but affect all individual hospitals (e.g., institutional environment, macroeconomic conditions, etc.). The specific model is set as follows:

(3) $$TE_{it}=\alpha +\beta D_{it}+\delta X_{it}+A_{i}+B_{t}+\varepsilon_{it}$$

where i=1,…,92 (number of hospitals in counties); t=2014,…,2018 (time in years); $TE_{it}$ represents the medical service efficiency of the i county hospital in t year. The binary variable $D_{it}\;$indicates county-level public general hospitals, and its value is 1 in the year and subsequent years when they participate in the CMC reform; otherwise, it is 0 [30,31,32]. Coefficient β measures the impact of CMC reform on the efficiency of medical services delivered by the county-level public general hospitals. If β is significantly positive, it indicates that the CMC reform has improved the efficiency of medical services. If β is significantly negative, it indicates that CMC reform has reduced the medical services efficiency of county-level public general hospitals. In addition, $A_{i}$ and $B_{t}$ are the dummy variables of hospitals’ individual and time fixed effects, respectively, while $X_{it}$ represents other control variables that affect the efficiency of medical services, and $\varepsilon_{it}$ represents random interference items.

Based on references to the existing literature [33,34,35,36,37], the factors that affect medical service efficiency in medical institutions can be divided into two categories, i.e., environmental and hospital-level factors. The environmental factors comprise regional population, regional economy, regional health resources, and reform policies. Hospital-level factors include medical services, hospital economic status, hospital resources (human and material), hospital management mechanism, patient factors, and hospital characteristics. Combining the characteristics and data availability of county-level public general hospitals in this study, eight indicators were selected as control variables. The definition and explanation of these variables are given in Table 2.

### 3.2. Data

**Figure 1:**
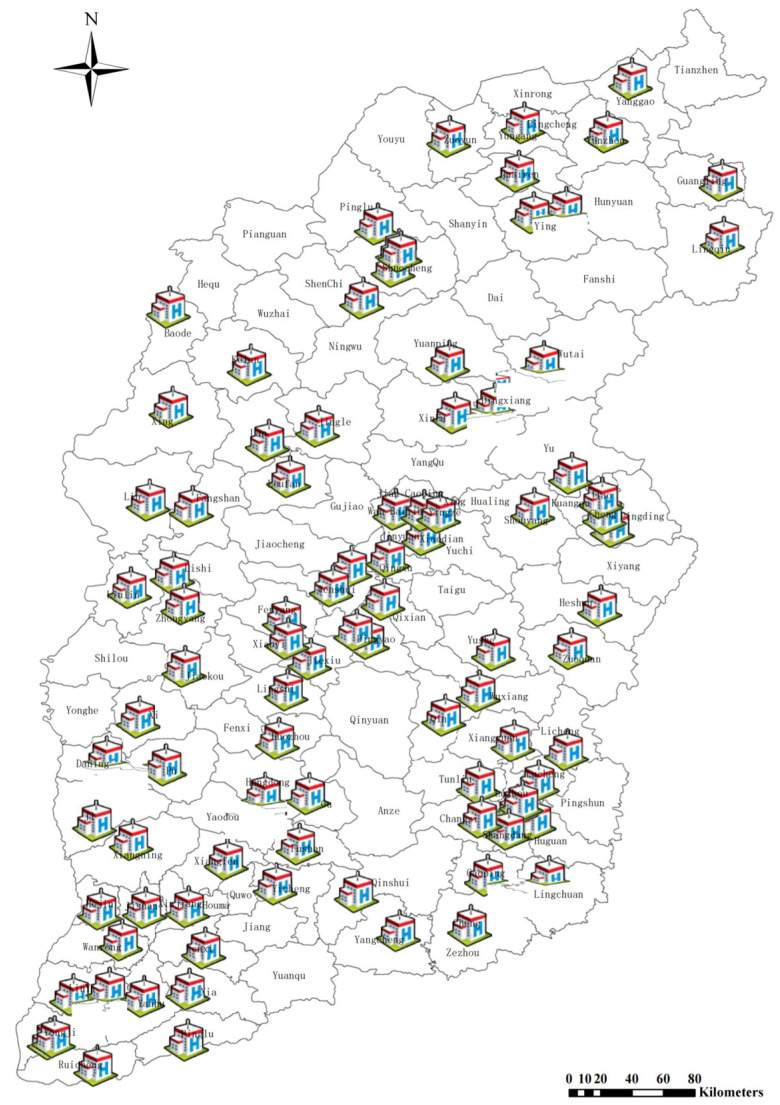
Spatial distribution of sample county-level public general hospitals in Shanxi Province.

The study’s sample was obtained from the Health Statistics Report published by the Provincial Health Commission in Shanxi province, China, for the period of 2014–2018 [38]. The Health Statistics Report provides statistics for a total of 92 county-level public general hospitals from 92 counties. Based on the development of economic, social, and health resource conditions in the county where the hospitals are located, they can be divided into three regions, viz., southern, central, and northern. The southern region comprises 43 counties, the central region 32 counties, and the northern region 17 counties. Spatial distribution of the 92 county-level public general hospitals in Shanxi Province is shown in Figure 1, with the column height representing the building area of each hospital.

## 4. Results

### 4.1. Input and Output of County-Level Public General Hospitals

The descriptive statistics (mean and standard deviation) of inputs and outputs variables are revealed in Table 3. On average, the inputs of licensed physicians, registered nurses, beds, and medical equipment increased from 2014 to 2018 by 8.7%, 21.3%, 19.7%, and 8.6%, respectively. The number of registered nurses increased the most, followed by the number of beds. The same trend was observed for the outputs of outpatient visits, emergency visits, discharged patients, and surgical patients from 2014 to 2018. They increased by 48.4%, 26.4%, 31.5%, and 44.6% during 2014–2018, with the number of outpatient visits and surgical patients showing the largest growth.

**Figure 2:**
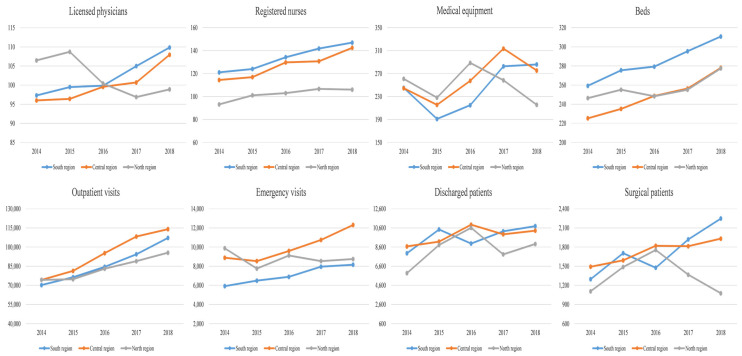
Changes in inputs and outputs of southern, central, and northern regions in Shanxi Province during 2014–2018.

Figure 2 shows the changes in the input-output of the southern, central, and northern regions in Shanxi Province during 2014–2018. The southern region demonstrated a relatively obvious trend of increasing input and output. The central region showed an increase in the inputs of licensed physicians, registered nurses, and beds, whereas the medical equipment showed a trend of rising first and then falling. The outputs of the central region also showed an increasing trend. In the northern region, the input of licensed physicians and medical equipment decreased, while the input of registered nurses and beds increased. In terms of output changes, the outpatient visits and discharged patients increased, whereas the emergency visits and surgical patients obviously decreased.

### 4.2. Medical Service Efficiency of County-Level Public General Hospitals

**Figure 3:**
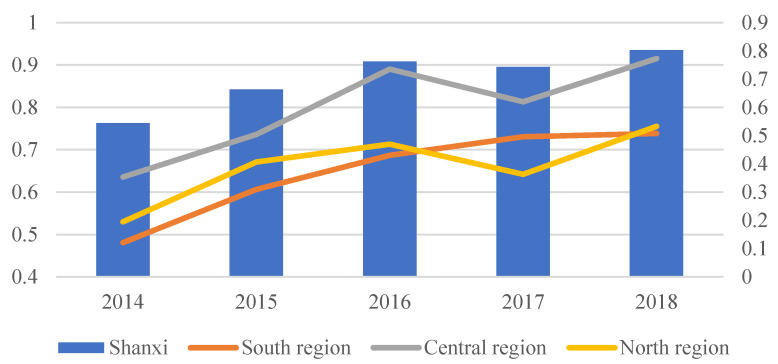
Medical service efficiency of county-level public general hospitals during 2014–2018.

Figure 3 indicates the medical service efficiency of county-level public general hospitals during 2014–2018. The results reveal that the medical service efficiency of county-level public general hospitals has generally increased. The medical service efficiency of all sample hospitals increased from 0.5439 in 2014 to 0.8032 in 2018. By comparing the changes in hospital medical service efficiency pre- and post reform (see Table 4), it can be observed that medical service efficiency before the reform was 0.6036, while after the reform, it rose to 0.7695.

Further comparison of the trends in medical services efficiency of hospitals before and after the reform reveals that the medical service efficiency of county-level public general hospitals in the southern, central, and northern regions of the Shanxi Province improved by varying amounts. For instance, the medical service efficiency of county-level public general hospitals in the southern region increased from 0.5436 to 0.7186 after the reform, while the medical service efficiency in the central region increased from 0.6859 to 0.8730, and the medical service efficiency in the northern region increased from 0.6006 to 0.7036 after reform, as seen in Table 4.

A paired-sample *t*-test was analyzed, and it shows that the medical service efficiency average score post-reform was more significant than that of the pre-reform period and was statistically significant at the 1% level. The same result was found in the southern, central, and northern regions at the 1% significance level (see Table 4).

### 4.3. Impact of the CMC Reform and Control Variables on the Medical Service Efficiency

The results of causal analysis between CMC reform and medical service efficiency are shown in Table 5 and Table 6. Models (1) and (2) in Table 5 provide the results for the overall county-level public general hospital sample estimation. Table 6 shows the results of the sub-sample policy effect evaluation model in the southern, central, and northern regions of Shanxi Province. Models (3), (4), (5), (6), (7), and (8) are the estimated results of the sample hospitals in different regions. Since the sample data used in this paper are panel data and contain the characteristics of cross-sectional and timeseries data, heteroscedasticity, cross-sectional autocorrelation, and serial autocorrelation tests were applied. According to the results in Table 5, model (1) has heteroscedasticity, cross-sectional autocorrelation, and serial autocorrelation. In Table 6, models (2), (6), (7), and (8) have heteroscedasticity and serial autocorrelation but do not have cross-sectional autocorrelation. Further, models (3), (4), and (5) only have heteroscedasticity. Therefore, in this paper, we adopted the clustering standard error estimation in models (1), (2), (6), (7), and (8), and the robust standard error estimation was used in models (3), (4), and (5) [39].

The results of the benchmark model (1) revealed that the variable CMCR is significant at the 1% level, demonstrating that the CMC reform improved the medical service efficiency of county-level public general hospitals. By including the control variables in the benchmark model, results show that the variable CMCR still had a significant impact on the medical service efficiency of county-level public general hospitals. The results of model (2) reveal that the medical service efficiency of county-level public general hospitals after the reform increased by 0.1568 compared with that experienced before the reform.

Moreover, other control factors also have a significant impact on the efficiency of county-level public general hospitals. Fixed assets, regional health centers, GDP, and population are positively and significantly associated with medical service efficiency at the 5% significance level, and urbanization rate was found to be significant and positively correlated at the 10% significance level.

Models (3) and (4) show the impact of the CMC reform on the medical service efficiency of county-level public general hospitals in the southern region. The results divulge that, regardless of whether the control variables are included or not, the CMC reform significantly impacted the medical service efficiency by 5%. The findings demonstrate that CMC reform has a positive effect on the efficiency of the medical services delivered by county-level public general hospitals in the southern region. The results in Table 6 show that the medical service efficiency of public hospitals in the southern region increased by 0.2190 after the reform. Models (5) and (6) reveal the impact of the CMC reform on the medical service efficiency of county-level public general hospitals in the central region. The study results show that without considering the control variables, the CMC reform has a positive impact on the medical service efficiency of the hospitals. Further, by adding the control variables, the impact becomes insignificant. Models (7) and (8) show the impact of the CMC reform on the medical service efficiency of county-level public general hospitals in the northern region. The results reveal that the medical service efficiency of public hospitals in the north region improved by 0.1329 (at 5%) after the reform.

The control factors also have a significant impact on the efficiency of county-level public general hospitals across regions. In the southern region, hospital stay is negatively associated with medical service efficiency at the 1% significance level, and regional health center is positively correlated at the 10% significance level. In the central region, regional health center, government subsidies, and GDP are positively and significantly associated with medical service efficiency. In the northern region, only the urbanization rate was found to have a positively significant impact on the medical service efficiency.

## 5. Discussion

### 5.1. Medical Service Efficiency Analysis

This study found that the medical service efficiency of county-level public general hospitals experienced a rising trend during 2014−2018. The same trend can also be observed in the southern, central, and northern regions. More importantly, this study proved the improvement of medical service efficiency in county-level public general hospitals pre- and post reform. The improvement of medical service efficiency can also be found in the southern and central regions. By further comparing the changes in input and output indicators of medical service efficiency in county-level public hospitals before and after the reform, the reason for the improvement of the efficiency can be seen.

Firstly, the supply of medical resources, such as human resources, beds, and equipment to the hospitals, increased after the reform, which was also found in previous studies [40,41]. From the sample statistics, we can observe that the number of licensed physicians and registered nurses employed in county-level public general hospitals after the reform increased by 8.7% and 21.3%, respectively, while the actual number of beds and the number of equipment also increased compared with before the reform. Secondly, the attraction of county-level public general hospitals to county citizens increased after the reform [4]. From our sample, we can observe that after the reform, the total number of outpatient visits in county-level public general hospitals increased by 48.4%, and the total number of surgical patients also increased by 44.6%.

### 5.2. The Impact of CMC Reform on Medical Service Efficiency

The aim of the CMC reform was to improve the operational efficiency of the three-level primary medical service system of county, township (town), and village through the integration of medical resources [5,42]. Our findings confirm the causal relationship between the CMC reform and county-level public general hospitals. Specifically, after controlling for other factors that affect the efficiency of hospital medical services, the county-level public general hospitals increased their medical service efficiency by 15.68% after joining the CMC; this is consistent with the results of Yuan et al. [21]. Moreover, the results of this study showed that the CMC reform had regional heterogeneity in its impact on the efficiency of county-level public general hospitals. Specifically, the medical service efficiency of hospitals in the southern and northern regions increased by 21.90%, and 13.29%, respectively, after the reform. It means that the CMC reform improved the medical service efficiency of county public general hospitals in the southern region more than in the northern region; Guo et al. [40] and Wei et al. [41] also reached similar conclusions. Although the medical service efficiency of public general hospitals in the central region was also improved by the reform, this causal relationship is not significant.

At present, many CMC reforms in China are still in the integration phase, focusing on the expansion of the medical community, but they lack internal management capabilities, such as incentivization systems, benefit-distribution mechanisms, and performance evaluation mechanisms [43]. Therefore, for areas with better economic development and high medical service efficiency, the expansion of the medical community will increase the redundancy of input and insufficient output, which in turn will hold back, to some extent, improvements in the medical service efficiency of county-level public general hospitals. Conversely, in areas with poor economic development and low medical service efficiency arising from a lack of medical resources, county medical community reform can expand the scale of medical institutions and increase the allocation of human, financial, and material resources within the medical community by integrating medical resources within the system. To a certain extent, the efficiency of hospital medical services has improved, while the degree of improvement is better than that of economically better-off areas.

The southern, central, and northern regions of Shanxi Province represent three different types of regions. The southern region was characterized by low economic development level and low medical service efficiency, with a per capita GDP of CNY 27,614 [44] and medical service efficiency of 0.5436 before the reform; the central region had the characteristics of high economic development level and high medical service efficiency, with a per capita GDP of CNY 34,939 [44] and medical service efficiency of 0.6859 before the reform. The northern region had the characteristics of high economic development level and medium medical service efficiency with a per capita GDP of CNY 35,633 [44] and medical service efficiency of 0.6006 before the reform. Therefore, the reform of the CMC had a greater impact on the southern region, followed by the northern region, with no significant impact on the central region.

### 5.3. The Impact of Control Variables on Medical Service Efficiency

The influence of the CMC reform on medical service efficiency went down after the control variables were included, which highlighted that the medical service efficiency of county-level public general hospitals is greatly affected by control variables.

Results showed that factors such as fixed assets, regional health center, GDP, population, and urbanization rate were positively and significantly related to medical service efficiency. From the regional perspective, the factors that affected the efficiency of medical services in different regions were not the same. The main factors in the southern region were hospital stay and regional health center; the main factors in the central region were regional health center, government subsidies, and GDP, while the only factor in the northern region was urbanization rate.

The CMC reform is a comprehensive reform, which is closely related to environmental and hospital-level factors. For environmental factors, the regions with higher economic levels are more attractive to high-quality medical human resources, while the hospitals in these regions find it easier to introduce talent and increase equipment, thus improving the performance of the hospitals [45]. Government subsidies are key to the operation of county-level public general hospitals. County-level government with strong financial strength is more capable of providing preferential policies and substantial financial support for the development of county-level public hospitals so as to improve hospital efficiency [35]. Furthermore, regions with a large population and high urbanization rates may have more medical needs and higher utilization of medical resources; thus, they may achieve better medical efficiency [46,47]. For hospital-level factors, the fixed assets and hospital stay are closely related to the input of doctors, nurses, beds, and equipment. The CMC is composed of three levels of medical institutions at the county, town, and village levels. However, due to different medical service organizations having differences in management concepts, performance appraisal principles, salary standards, hospital culture, and risk-control capabilities [44], it is necessary to allocate medical resources reasonably within the county medical community.

## 6. Conclusions and Limitations

### 6.1. Conclusions 

This paper selected 92 county-level public general hospitals in Shanxi Province in central China as the research objective, using the super efficiency SBM-DEA model and two-way fixed-effect model to study the impact of the CMC reform on the medical service efficiency of county-level public general hospitals based on the data obtained from the Health Statistics Report published by the province’s Health Commission for the years 2014–2018. The study found that the CMC reform improved the medical service efficiency of county public general hospitals by 15.6% in Shanxi Province. The CMC reform had regional heterogeneity in its impact on the efficiency of county-level public general hospitals. The CMC reform improved the medical service efficiency of county public general hospitals in the southern region more than in the northern region. Medical service efficiency in the central region was also improved by the reform, but the causal relationship is not significant. Hospital-level factors such as fixed assets, hospital stay, and regional health center and environmental factors such as GDP, population, urbanization rate, and government subsidies affected the medical service efficiency of county public hospitals in promoting the construction of CMC.

These results not only present policy implications for Shanxi Province but also broader policy implications for many regions in China and also global regions that face significant integration issues in terms of medical resources in rural areas. The empirical results of this study highlight three key policy implications. First, county-level governments should further promote CMC reform to improve the medical service efficiency of county-level public general hospitals, thereby improving the operational efficiency of the three-level primary medical and health service system. Second, county-level governments should pay greater attention to the economic conditions and medical resource levels of different regions during the process of promoting CMC and formulate precise policies based on specific regional conditions. For instance, in areas with good economic development and medical resources, the focus should be on improving management efficiency within CMC and the operational efficiency and resource utilization of medical institutions. Conversely, in areas with poor economic development and medical resources, the focus during the construction of CMC should be on expanding the size of that community by increasing the total amount of medical resources with a better allocation of medical resources in the short term; this will encourage the improvement of the internal management efficiency of the medical community in the long term. Third, some environmental factors such as economic, government, and population characteristics should be allowed for within the policy of promoting the CMC reform in the future.

### 6.2. Limitations

Although this study explained the deepest investigation regarding efficiency of county-level public general hospitals, due to lack of data availability, this article has a few limitations. First, the samples studied in this paper are from the central China, and therefore, there is a lack of sample data from other regions of the country. Second, there are some missing factors due to the availability of data, for instance, hospital staff salary. Third, this paper mainly focused on determining whether county medical community reform had improved the medical service efficiency of county public general hospitals; however, it could pay further attention to the mechanism of the impact of CMC reform on the medical service efficiency of county public general hospitals and compare with other provinces.

## Figures and Tables

**Table 1:** Description of Efficiency Evaluation Model Variables.

	Variable	Explanation of Variable
Input variables	Licensed physicians	Total number of licensed physicians employed by the county-level public general hospitals
	Registered nurses	Total number of registered nurses employed by the county-level public general hospitals
	Beds	Number of fixed beds at the end of the year in the county-level public general hospitals
	Medical equipment	Number of medical equipment above ten thousand RMB in the county-level public general hospitals
Output variables	Outpatient visits	Total number of registered outpatients in the county-level public general hospitals
	Emergency visits	Total number of registered persons in emergency department in the county-level public general hospitals
	Discharged patients	Number of persons discharged from all county-level public general hospitals after hospitalization
	Surgical patients	Total number of inpatients undergoing operations and procedures in the county-level public general hospitals

**Table 2:** Description of Policy Effect Evaluation Model Variables.

	Variable	Explanation of Variable
Explained variable	Medical service efficiency	Calculated by the efficiency evaluation model
Core explanatory variable	County medical community reform (CMCR)	Expressed as the value of the county-level public general hospitals in the year and subsequent years of participating in the CMC reform is 1; otherwise, it is 0
Control variables	Employees (Emp)	Total number of employees in the county-level public general hospitals
	Fixed assets (FA)	Total fixed assets/Total number of medical technicians
	Hospital stay (HS)	Average length of hospital stay in the county-level public general hospitals
	Regional health center (RHC)	Whether or not the county-level public general hospital is a regional health center
	Government subsidies (GS)	Revenue from fiscal subsidies of the county-level public general hospital
	GDP	Per capita GDP of the county where the county-level public general hospital is located
	Population (Pop)	Total population of the county where the county-level public general hospital is located
	Urbanization rate (UR)	Proportion of urban population of the county where the county-level public general hospital is located

**Table 3:** Descriptive statistics of input and output variables.

Variable		2014		2015		2016		2017		2018	
		Mean	S.D.	Mean	S.D.	Mean	S.D.	Mean	S.D.	Mean	S.D.
Input variables	Licensed physicians	98.54	53.53	100.12	53.62	99.86	49.51	101.99	49.89	107.15	54.39
	Registered nurses	113.62	71.38	117.34	68.67	126.91	72.74	131.48	77.52	137.87	87.14
	Beds	245.09	126.64	257.72	132.41	262.95	139.59	274.41	147.06	293.27	154.49
	Medical equipment	247.87	122.23	206.43	142.07	243.38	149.35	288.93	172.21	269.26	133.05
Output variables	Outpatient visits	72,355.25	50,216.88	77,762.86	50,657.16	87,947.82	53,513.51	98,160.49	59,702.18	107,394.1	61,543.2
	Emergency visits	7679.63	7350.82	7431.01	5644.81	8237.75	6300.79	9026.80	6863.66	9707.02	7376.06
	Discharged patients	7815.45	5553.82	9693.62	4114.84	9953.52	5853.33	9689.90	5023.67	10,273.35	5787.43
	Surgical patients	1328.76	1345.24	1624.29	1250.88	1646.65	1279.46	1780.25	1072.87	1920.76	1598.51

**Table 4:** Medical service efficiency before and after the CMC reform.

	Pre-Reform (2014~2015)		Post-Reform (2016~2018)		Pre-Reform—Post-Reform	
	Mean	S.D.	Mean	S.D.	*t*-Value	*p*-Value
Shanxi	0.6036	0.4259	0.7695	0.3841	−5.336	0.000
Southern region	0.5436	0.3895	0.7186	0.3617	−3.315	0.002
Central region	0.6859	0.4561	0.8730	0.4056	−4.264	0.000
Northern region	0.6006	0.4420	0.7036	0.3640	−4.477	0.000

**Table 5:** Results of the overall sample policy effect evaluation model.

Variable	Medical Service Efficiency	
	(1)	(2)
CMCR	0.2594 ***	0.1568 ***
	0.0490	0.0108
Emp		0.0002
		0.0001
FA		0.0753 **
		0.0208
HS		0.0004
		0.0010
RHC		0.1632 **
		0.0385
GS		0.0134
		0.0117
GDP		0.0265 **
		0.0084
POP		0.0097 **
		0.0027
UR		0.5946 *
		0.2382
Hospital FE	Y	Y
Year FE	Y	Y
Heteroscedasticity test (chi-square)	25,075.36 ***	64,836.85 ***
Cross-sectional autocorrelation test (Pesaran’s test)	2.51 **	0.28
Serial autocorrelation test (F)	5.41 **	6.31 **
R^2^	0.1005	0.1249
Sample size	460	460

Note: ***, **, and * in the table indicate significance levels of 1%, 5%, and 10%, respectively, and the brackets indicate standard error.

**Table 6:** Results of the sub-sample policy effect evaluation model in the southern, central, and northern regions.

Variable	Medical Service Efficiency					
	Southern Region		Central Region		Northern Region	
	(3)	(4)	(5)	(6)	(7)	(8)
CMCR	0.2577 ***	0.2190**	0.2797 ***	0.0682	0.2252 ***	0.1329 **
	0.0762	0.0889	0.0770	0.0872	0.1221	0.0308
Emp		0.0001		−0.0003		−0.0004
		0.0001		0.0002		0.0016
FA		0.0708		0.1372		0.0225
		0.1109		0.1015		0.1212
HS		−0.0988 ***		0.0006		−0.0131
		0.0322		0.0008		0.0232
RHC		0.1814 *		0.2621 ***		0.1421
		0.1021		0.0099		0.1312
GS		−0.0265		0.1703 ***		0.0343
		0.0530		0.0327		0.0620
GDP		−0.0010		0.0262 **		0.0328
		0.0107		0.0059		0.0464
POP		0.0018		0.0082		0.0115
		0.0014		0.0098		0.0072
UR		−0.2654		−0.0854		1.4819 *
		0.2156		0.7617		0.6857
Hospital FE	Y	Y	Y	Y	Y	Y
Year FE	Y	Y	Y	Y	Y	Y
Heteroscedasticity test (chi-square)	1912.68 ***	5447.98 ***	6460.12 ***	20,012.76 ***	17,714.09 ***	45,199.34 ***
Cross-sectional autocorrelation test (Pesaran’s test)	0.05	0.13	0.30	−0.11	0.05	−0.56
Serial autocorrelation test (F)	0.35	1.01	1.76	3.04 *	18.23 ***	25.88 ***
R^2^	0.1094	0.1690	0.1232	0.1802	0.075	0.1277
Sample size	215	215	160	160	85	85

Note: In the table, ***, **, and * indicate significance levels of 1%, 5%, and 10%, respectively, and the brackets indicate standard error.

## Data Availability

The data presented in this study are available on request from the corresponding author. The data are not publicly available for privacy and confidentiality reasons.
